# State-of-the-Art on Biomarkers for Anaphylaxis in Obstetrics

**DOI:** 10.3390/life11090870

**Published:** 2021-08-24

**Authors:** Anca Angela Simionescu, Ana Maria Alexandra Stanescu, Florin-Dan Popescu

**Affiliations:** 1 Department of Obstetrics and Gynecology Filantropia Clinical Hospital, Carol Davila University of Medicine and Pharmacy, 050474 Bucharest, Romania; asimion2002@yahoo.com; 2 Department of Family Medicine, Carol Davila University of Medicine and Pharmacy, 050474 Bucharest, Romania; 3 Department of Allergology Clinical Hospital Nicolae Malaxa, Carol Davila University of Medicine and Pharmacy, 022441 Bucharest, Romania; florindanpopescu@gmail.com

**Keywords:** anaphylaxis, pregnancy, biomarkers, tryptase, in vitro allergy tests, immunoassays

## Abstract

Anaphylaxis is an unpredictable systemic hypersensitivity reaction and constitutes a high risk of maternal and fetal morbidity and mortality when occurring during pregnancy. Currently, the acute management of anaphylaxis is based on clinical parameters. A total serum tryptase is only used to support an accurate diagnosis. There is a need to detect other biomarkers to further assess high-risk patients in obstetrics. Our objective is to present biomarkers in this complex interdisciplinary approach beyond obstetrician and anaesthetic management. Candidate biomarkers derive either from mediators involved in immunopathogenesis or upcoming molecules from systems biology and proteomics. Serum tryptase is determined by singleplex immunoassay method and is important in the evaluation of anaphylactic mast cell degranulation but also in the assessment of other risk factors for anaphylaxis such as systemic mastocytosis. Another category of biomarkers investigates the IgE-mediated sensitization to triggers potentially involved in the etiology of anaphylaxis in pregnant women, using singleplex or multiplex immunoassays. These in vitro tests with natural extracts from foods, venoms, latex or drugs, as well as with molecular allergen components, are useful because in vivo allergy tests cannot be performed on pregnant women in such a major medical emergency due to their additional potential risk of anaphylaxis.

## 1. Introduction

Anaphylaxis is an unpredictable, severe and potentially life-threatening hypersensitivity reaction [1,2]. Drug and food-related anaphylaxis has increased over time in parallel with an increased incidence of overall anaphylaxis from the last decade, according to data from Western countries [3,4]. Anaphylaxis during pregnancy has the potential to cause devastating human damage, and the majority of adverse outcomes frequently affect both, mother and the unborn baby and neonate. Therefore, anaphylaxis in pregnancy remains a maternal mortality related cause [5,6]. 

Incidence of anaphylaxis in pregnancy seems similar across European countries, affecting 1.5 per 100,000 women among almost 4.5 million births, the majority of these hypersensitivity reactions occurring at or around the time of birth. A prospective study from the United Kingdom reported 1.6 cases per 100,000 pregnancies. Retrospective studies have found rates of 2.6 to 3.8 per 100,000 pregnancies [6,7]. 

Currently, the acute management of anaphylaxis is based on clinical parameters. Manifestations of anaphylaxis include skin and mucosal involvement (e.g., generalized urticaria, pruritus or flushing, swollen lips-tongue-uvula), respiratory compromise (e.g., dyspnea, wheeze-bronchospasm, stridor), gastrointestinal symptoms (e.g., severe crampy abdominal pain, repetitive vomiting), reduced blood pressure or associated symptoms of end-organ dysfunction (e.g., hypotonia/collapse, syncope, incontinence). These symptoms have an acute onset, minutes to several hours, after exposure to a known or highly probable trigger. Severe anaphylaxis may occur without typical findings as skin features or circulatory shock [2,8,9]. 

Because it is a life-threatening condition for mothers with potential neurologic sequels for fetuses, anaphylaxis during pregnancy require timely management. A total serum tryptase is used to support the diagnosis and differentiate from other causes of hypotension in pregnancy. There is a need for more validated decisional biomarkers to further assess this high-risk population, which necessitate additional exposure to potential anaphylactic triggers during obstetrical procedures. Here, we present current biomarkers effective for targeting the high-risk population as well as for the prognostic severity. Our objective is to present and highlight the usefulness of biomarkers in this complex interdisciplinary approach beyond obstetrician and anaesthetic management.

## 2. Clinical Diagnosis of Anaphylaxis 

Diagnosis of anaphylaxis is a clinical one. These symptoms have an acute onset, minutes to several hours after exposure to a known or highly probable trigger. The most frequent triggers for anaphylaxis are drugs, foods and insect venoms [8]. 

The World Allergy Organization has been elaborating an updated and comprehensive guideline and proposed clinical criteria for diagnosing anaphylaxis, a valuable aid for timely diagnosis [8,9] (Table 1). 

Anaphylaxis may induce maternal hypotension and hypoxemia, potentially life-threatening to both mother and fetus. Maternal hypotension and vasoconstriction can result in decreased uterine blood flow, and maternal hypoxemia can lead to intrapartum asphyxia [10,11,12,13]. 

Hypotension may be the most objective sign of anaphylaxis. As a result, anaesthetists and obstetricians should be aware of other causes of hypotension in pregnant or postpartum women [14].

Other common pregnancy-related manifestations which can also be present in anaphylaxis are lower back pain, uterine cramps, vulvar and/or vaginal pruritus, presumably due to mast cell degranulation in the vagina and uterus, rich in these cells [13,15]. 

Fetal risks include fetal distress and preterm labour, hypoxic-ischemic encephalopathy, severe central nervous system damage, or even death [12,16].

## 3. The Immunology of Anaphylaxis and Pregnancy-Brief Overview

The etiology of anaphylaxis is very diverse and frequently unexpected. Anaphylaxis can be determined by immunologic or non-immunologic mechanisms [17]. The complexity of the pathogenesis of anaphylaxis throughout pregnancy is attributed to specific immune tolerance during pregnancy and immune response mechanisms interference, a dynamic heterogeneous combination of hyperresponsiveness and inflammatory responses [18]. During the first trimester, etiologies are similar to those in nonpregnant women, while during labor and delivery, possible etiologies include beta-lactam antibiotics and other agents commonly used in medical and perioperative settings [19,20]. During pregnancy, the maternal immune system undergoes various changes to tolerate the product of conception. Because of this, cytokine signaling, suppression of the T cell responses, or alteration of cell-mediated immunity lead to a higher risk of pregnant women to have an abnormal immune response [21]. Among other significant changes in humans and murine models are the increase in Th2-type cytokine production and the inhibition of cytokine production by Th1 cells [21,22,23]. Pregnancy is characterized by immune shifts to facilitate maternal-fetal tolerance, with maternal immune response changes from the inflammatory Th1 cytokine pattern to the Th2 pattern [24].

From an obstetrical approach, it is also important to mention that mast cells are largely present within the uterus and placenta. Postpartum examination of the placenta reveals massive degranulation of mast cells and severe vasoconstriction of umbilical arteries. In an isolated cotyledon, histamine induces contraction by activating H_1_ receptors and secondary relaxation of placental chorionic plate arteries. Umbilical vessels being not innervated, the flow control depends on vasoactive mediators either locally released or from the circulation [25]. Moreover, mast cells are overactive in recurrent pregnancy losses by creating a pro-inflammatory milieu, suggesting a novel role in the immunopathology. Future studies are needed to better understand their role in implantation and placental angiogenesis [26].

Possible additional pathogenetic hormonal mechanisms were suggested: withdrawal of the stabilizing effect of progesterone on mast cells, together with influences of prolactin, oxytocin, adrenocorticotropic hormone and corticotropin-releasing hormone [27]. 

The immunologic IgE-mediated mechanism involves a sensitization process including the activation of Th2 cells by the triggers, with the induction of specific IgE production. These IgE molecules bind to Fc*ε*RI receptors on mast cells, basophils, or both. This determines a complex intracellular signaling cascade that leads to degranulation and the immediate release of preformed mediators such as histamine and tryptase, carboxypeptidase A, and proteoglycans [28,29,30]. The cascade of phospholipase A2, cyclooxygenase, lipoxygenase, and platelet activating factor (PAF) is activated, with the later release of inflammatory cytokines and chemokines.

Bruton’s tyrosine kinase is an essential kinase for signaling through the high-affinity receptor Fc*ε*RI on the surface of mast cells and basophils, the activated signaling cascade causes the release of numerous allergic mediators including histamine, prostaglandins, leukotrienes, and cytokines responsible for inducing signs and symptoms in anaphylaxis [31].

The immunologic Th2 shift in pregnancy, similar to allergic responses, causes an increase in the production of specific cytokines, IL-4, IL-5, IL-6, and IL-13, which promote B cell expansion and antibody class switching. Total serum IgE and IL-4, a cytokine that stimulates class switching to IgE, are elevated during pregnancy. The maternal-fetus interface is responsible for immunological protection and allergen mediated responses. Immune responses during early pregnancy are different from those in mid and late pregnancy. More important, a cellular immunological imbalance of the decidual immune system, with the predominance of T cells and dendritic cells (DC), is associated with recurrent spontaneous abortion, pre-eclampsia, preterm birth, intrauterine growth restriction, and infection [19,24,32,33]. 

Higher levels of histamine and IL-4 have been reported in the serum of human patients with severe anaphylaxis, indicating that these molecules may be involved in the expression of the severe disease phenotype. IL-4 amplifies IgE- and histamine-induced endothelial dysfunction, fluid extravasation, and the severity of anaphylaxis [34,35].

Most human cases of anaphylaxis are IgE-mediated. However, there is an increasing number of publications supporting the direct stimulation of mast cell degranulation, including activation of the Mas-related G protein-coupled receptor MRGPRX2, IgG and complement involvement and other non-IgE-mediated mechanisms of anaphylaxis for certain triggers [2,17,36,37,38,39,40]. In the Table 2 are presented the most important anaphylaxis triggers reported in pregnancy, with selected references regarding the listed ones, together with classical immunologic or nonimmunologic mechanisms involved, associated effector cells and important mediators, as described mainly in non-obstetrical patients [1,2,17,19,20]. Anaphylaxis after intravenous iron administration in pregnant women, even fatal, was reported. Serum tryptase levels may be elevated in such a severe hypersensitivity reaction involving complement activation-related pseudoallergy [41,42,43,44]. More detailed information on anaphylaxis triggers, such as drugs and latex, in pregnant women is presented in the following biomarker chapter. 

Allergic anaphylactic reactions are triggered when allergens enter the blood circulation and activate immunoglobulin E (IgE)-sensitized extravascular mast cells, causing systemic immediate discharge of prestored proinflammatory mediators. Although perivascular macrophages are capable of transendothelial sampling slowly after exposure to blood-borne antigens, these are rapidly adsorbed by the lamellipodia of CD301b^+^ perivascular dendritic cells that protrude through the endothelial wall into the vessels. Such cells continuously sample blood and spontaneously shed antigen-bound microvesicles (generated by vacuolar protein VPS4) to neighboring IgE-bearing mast cells in the perivascular region which vigorously degranulate and trigger anaphylaxis [45]. 

## 4. Biomarkers 

Biomarkers are indicators of biological or pathogenic processes or responses to an exposure or intervention, which possess properties that allow their objective measurements in biological samples [2,46,47,48]. There is a constant need to translate from biomarker discovery to clinical utility in predictive and personalized medicine. An ideal biomarker for anaphylaxis is not discovered yet, but it should be highly specific, sensitive, predictive, with good in vivo and in vitro stability, quick and easy to assess, noninvasive and not expensive. 

At present, several molecular biomarkers can be used in the diagnosis of anaphylaxis in acute care settings and to assess later on the trigger involved, but there are many unmet needs according to experts [49]. 

### 4.1. Mast Cell Tryptase 

In a population-based multinational European study, from 28 cases of anaphylaxis in pregnancy in France, Belgium and Finland, serum tryptase was tested in 53.8%, levels being raised in 84.6% of cases, compared with 35 cases of anaphylaxis in pregnancy in UK in which serum tryptase was tested in 85.7%, with increased levels in 32% of cases [6]. Therefore, a normal total tryptase level cannot be used to refute the diagnosis of anaphylaxis in pregnancy. In some patients with anaphylaxis, such as those with food-triggered anaphylaxis or the patients normotensive, elevations in serum tryptase generally do not occur [19,50]. 

**Figure 1:**
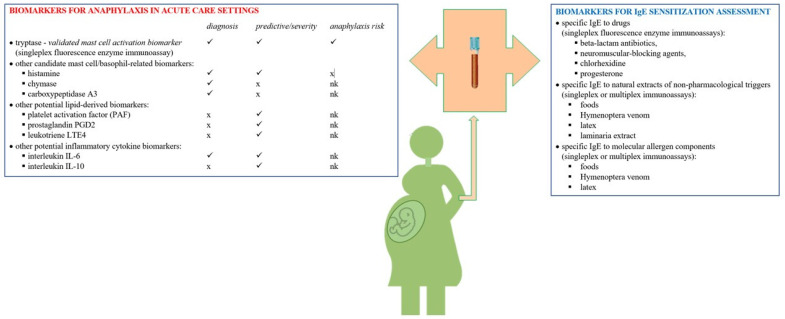
Biomarkers for anaphylaxis in pregnancy. Legend: ✓ = role known, x = not demonstrated; nk = not known; data adapted from [51].

Tryptase is the most abundant protein in mast cells. Mature *β*-tryptase is stored in mast cell granules and released on activation in anaphylaxis, whereas *α*- and *β*-protryptases are secreted constitutively by mast cells. Total tryptase consists of an immature monomer isoform (continuously but weakly released in serum by mast cells) and a heterotetramer mature isoform (suddenly and rapidly released upon mast cell degranulation). This protease is the validated mast cell activation biomarker assessed during the acute management of anaphylaxis. Other candidate biomarkers are mast-cell- basophil-related, lipid-derived and inflammatory cytokines [51,52,53,54,55] (Figure 1).

Tryptase is considered a mast cell-derived product, being present in much lower amounts in basophils. At the onset of anaphylaxis, tryptase is released from mast cells alongside other mediators. Compared to histamine, mast cell tryptase has a longer plasma half-life of approximately 2 h; therefore it is the clinically introduced biomarker of anaphylaxis. During acute anaphylaxis, serum tryptase levels are increased from 15 min to 3 h or even longer, after the onset of symptoms; its levels peak between 1 and 2 h after the onset, but in about 35–40% of patients remains less than 11.4 μg/L [2,8,52,53,54,55]. 

The quantitative measurement of total tryptase in human serum or plasma is performed by fluorescence enzyme immunoassay with capsulated cellulose polymer solid-phase. This method determines the total tryptase levels including all forms of α-tryptase and *ß*-tryptase. Its principle consists in the reaction of the tryptase in the patient sample with anti-tryptase covalently coupled to the solid phase consisting of a cellulose derivative enclosed in a capsule, *ß*-galactosidase-labeled antibodies against tryptase are added to form a complex incubated with a developing agent, 4-methylumbelliferyl-*ß*-D-galactoside, with fluorescence measurement. The total time for one singleplex immunoassay is 2.5 h. Blood samples for the tryptase singleplex immunoassay should be collected by venipuncture. Serum or plasma (EDTA, lithium heparin or sodium heparin) samples from venous blood can be used. The first blood sample should be taken no earlier than 15 min from the onset and up to 3 h after the onset of the acute reaction causing mast cell activation (usually between 30 min and 3 h), and the time between the reaction and sample collection must be noted. Elevated tryptase levels can be detected up to 6 h following an anaphylactic reaction and return to baseline levels 24 h after release. A very early sampling time (<30 min) may potentially generate a false negative result. Two early sampling times may be recommended, first as soon as possible and the second one at 1–2 h, but no later than 4–6 h after the onset of symptoms, but sometimes this may be challenging with in a busy emergency department. Another sample for baseline values should be taken at least 24 h after resolution of symptoms. Specimens may be kept at room temperature for shipping purposes for 2 days, stored at 2–8 °C if assayed within 5 days after collection, while for more extended periods, storage is to be at −20 °C or −70 °C. The measuring range for total tryptase is 1–200 µg/L (for higher values diluting the samples is needed), and only 40 µL of serum or plasma is needed per test. Other 30–100 µL of serum or plasma are needed for multiplex immunoassays to assess IgE sensitization profiles [56,57]. 

Accordingly, it is recommended to reevaluate serum tryptase at baseline, at least 24 h after resolution of anaphylaxis symptoms, even when tryptase concentration during episode remains within normal range. A cut off of 20 μg/L constitutes one minor WHO criteria for systemic mastocytosis. An elevated baseline tryptase concentration may uncover systemic mastocytosis, a rare disease in which mast cells infiltrate extracutaneous tissues, with an increased risk of anaphylaxis which may be its first manifestation [8]. A “20% + 2 consensus equation” was validated in clinical practice and is currently considered significant as a criterion of severe acute mast cell activation, meaning that peak mast cell tryptase should be more than 1.2 *×* baseline tryptase + 2 ng/mL [58,59]. Moreover, high serum tryptase is a promising forensic biomarker for deaths due to anaphylactic shock in general, especially when it is higher than 30.4 μg/L [60]. A recent systematic review found that the mentioned equation is underused in the diagnosis of anaphylaxis. Serial tryptase or paired measurements are unfortunately frequently considered laborious and with limited application in daily clinical practice. In selected cases, the tryptase ELISA B12 immunoassay for *α*-tryptase and *β*-tryptase performed in conjunction with a biotin-G5 immunoassay for *β*-tryptase, provides a precise measure of mast cell involvement [61]. 

Elevated basal serum total tryptase (above 8 ng/mL) with normal mature tryptase levels (<1 ng/mL) is a defining feature of hereditary *α*-tryptasemia, an autosomal dominant genetic trait found in 4–6% of the general population and defined by excess copies of *α*-tryptase at TPSAB1 with an increased pro-*α*-tryptase synthesis and secretion. In this condition, there is an increased incidence and severity of anaphylaxis [62]. Hereditary *α*-tryptasemia is a valid genetic biomarker in mastocytosis useful for determining the individual risk of developing severe anaphylaxis [63].

In general, any elevation in serum tryptase provides evidence of mast cell activation and thus supports a diagnosis of anaphylaxis in a suggestive clinical context. But it should be kept in mid that false increased tryptase levels, although uncommon, may be associated with the presence of heterophilic antibodies in serum, usually human antimouse antibodies. Patients treated with mouse-human chimeric monoclonal antibodies, such as infliximab, or patients with extensive exposure to mice, are at increased risk for having such antibodies. A solution to this problem is adding a heterophilic antibody suppressor to the tryptase immunoassay kit [64,65,66].

Elevated baseline total tryptase levels are rarely detected in the following disorders: acute myeloid leukaemia, myelodysplastic syndromes, myeloid variants of the hypereosinophilic syndrome, especially those associated with the Fip1-like1-platelet-derived growth factor receptor *α* [FIP1L1-PDGFRA] mutation, GATA-2 haploinsufficiency, severe renal failure and hemodialysis patients [67,68,69,70,71,72].

There are not many case reports of anaphylaxis in pregnancy in which serum tryptase or other biomarkers were performed. In a case of allergic anaphylaxis in late pregnancy due to amoxicillin, the plasma concentrations of histamine and tryptase performed at delivery by emergency cesarean section and 120 min later, in both the mother and neonate, revealed normal results (histamine less than 10 nmol/L, tryptase less than 11 μg/L) [25].

In a report of anaphylactic reaction due to rocuronium-sugammadex complex during elective cesarean section, the serum tryptase level was significantly elevated [73]. Serum tryptase level was reported increased also in anaphylactic shock after misoprostol in voluntary termination of pregnancy [74].

Elevated tryptase levels have also been reported in amniotic fluid embolism (AFE) [75]. AFE is an important cause of acute cardiovascular collapse during pregnancy and can be difficult to distinguish clinically from anaphylaxis. Suggested criteria for AFE syndrome include one or more of the clinical findings of sudden-onset cardiovascular collapse, respiratory distress, or disseminated intravascular coagulation during pregnancy and absence of other medical explanations for the clinical course. Disseminated intravascular coagulopathy (DIC) is a key laboratory abnormality occurring in most patients, with elevated D-dimer, low fibrinogen (especially <200 mg/L) and thrombocytopenia [76,77,78]. Large volume blood loss should raise suspicion for AFE, while bronchospasm is a characteristic of anaphylaxis rather than AFE. Coagulopathy and/or disseminated intravascular coagulation are more often associated with AFE, although activation of both the coagulation and contact systems can also appear in anaphylaxis [50,79]. AFE has been considered in the past “anaphylactoid syndrome of pregnancy”. The pathogenesis of this rare and typically catastrophic condition involves the entry of amniotic fluid, which contains fetal and other antigenic materials, into the maternal systemic circulation via a breach in maternal/fetal interface leading to abnormal activation of humoral and immunological processes and release of tryptase, histamine and other vasoactive or procoagulant substances, similar to the systemic inflammatory response syndrome. An elevated *β*-tryptase level was reported in a fatal case [75]. Complement activation has also been suggested as a possible mechanism. AFE pathophysiology suggests the importance of *β*-tryptase and complement fractions C3-C4 as diagnosis tools [75,80,81,82]. 

Tryptase levels may be increased also in the amniotic fluid. Blood tests for sialyl-Tn (STN) antigen may be helpful in AFE diagnosis, being a biomarker predictive for life-threatening or fatal episodes. The sialyl Tn structure, NeuAc alpha 2–6GalNAc alpha 1-O-Ser/Thr, recognized by monoclonal antibody TKH-2 is a characteristic component in meconium and amniotic fluid. The concentration of the STN antigen may be determined by an immunoradiometric competitive inhibition assay using the monoclonal antibody TKH-2 [79,83]. 

To sum up, total serum tryptase still remains the best current biomarker to support the diagnosis of anaphylaxis, elevated levels were also reported in AFE, and there is a need for further evaluation of other biomarkers in these life-threatening conditions [84].

### 4.2. Other Potential Biomarkers for Diagnosis of Anaphylaxis in the Acute Care Setting 

In an attempt to discriminate between patients with mastocytosis with or without anaphylaxis, in addition to plasma tryptase, three new proteins were identified as candidate biomarkers, pregnancy-associated plasma protein-A (PAPP-A), galectin-3, and allergin-1, with significantly different levels in patients with mastocytosis with anaphylaxis compared with those without anaphylaxis [85].

Histamine is also a classic biomarker of mast cell and basophil activation. In anaphylaxis, plasma histamine peaks within 5–10 min of the onset of symptoms and declines towards baseline within 30 min as a consequence of rapid metabolism by diamine oxidase (DAO) and *N*-methyltransferase. Important disadvantages for this biomarker are its short half-life (less than 15 min, approximately 2–3 min) and poor ex vivo stability, serum samples needing to be frozen promptly. *N*-methyl histamine, a stable end product of histamine, easier to quantitate, may be measured in urine by competitive enzyme-linked immunosorbent assay (ELISA), its levels being highly correlated with histamine in plasma [2,10,54]. However, bacteria in the digestive or urinary tract and histamine-rich foods have been reported to increase histamine metabolite levels [55].

In an uncomplicated pregnancy, a balance between histamine and histamine degradation by the enzyme DAO seems crucial. DAO activity, largely produced by the placenta, increases during normal pregnancy by up to 1000-fold and prevents excessive access of histamine into the fetal circulation. Therefore, maternal plasma histamine does not usually increase in the case of anaphylaxis. DAO is also present in the amniotic fluid. In an uncomplicated pregnancy, the concentration of DAO is elevated in the newborn’s serum at birth and it does not decline until after a week. The only data on plasma histamine concentrations in the normal neonate is related to umbilical cord blood, being below the lowest detection limit of 1.8 ng/mL of an immunoassay [25].

Histamine and other mediators of anaphylaxis, such as endothelin and bradykinin, produce appreciable contractions on human umbilical vessels, while thromboxane A2 and 5-hydroxytryptamine are considered to be physiologic mediators of umbilical artery closure [25]. 

Basogranulin is released from basophils in parallel with histamine, and this may be quantified by dot blotting. Basogranulin is a novel basophils granule protein recognized by the monoclonal antibody BB1. The role of this unique secretory marker of basophils, with maximal serum levels at 15 min in anaphylaxis as a suitable biomarker in anaphylaxis, is uncertain, only preliminary data being published [54,86].

Chymase and carboxypeptidase A3 are, alongside with tryptase, pre-formed mediators of anaphylaxis released by activated mast cells. Chymase is a serine protease from their secretory granules, potentially stable in serum, with the potential as a biomarker in anaphylaxis, its concentration remaining high at least 24 h after the onset of the reaction. Chymase levels in anaphylaxis are not correlated with the levels of mast cell tryptase, but with those of carboxypeptidase and dipeptidyl peptidase 1 (DPP1). DPP1, also known as cathepsin C, is expressed by numerous cell types including both mast cells and basophils and. Chymase levels measured by ELISA in postmortem serum were detected as elevated (>3 ng/mL) in fatal anaphylaxis. Carboxypeptidase A3, detectable in serum and saliva, has also a half-life longer than tryptase, this being a potential advantage if sampling time is later than 2 h from the onset of symptoms and in cases when mast cell tryptase level is not increased. Carboxypeptidase A3 levels were elevated (>14 ng/mL) in serum from patients with a clinical diagnosis of anaphylaxis, but not in healthy controls or those with atopy. Nevertheless, the role as anaphylaxis biomarkers of serum chymase and carboxypeptidase A3 remain experimental [54,87,88,89]. 

The major basophil chemotactic factor, chemokine (C-C motif) ligand 2 (CCL2), was also examined as a biomarker in anaphylaxis, but only preliminary data are published. A cut-off serum level of 334 pg/μL is mentioned. CCL2 serum levels may correlate with the severity of anaphylaxis, but glycosylation influences its chemotactic potency [54,90]. CCL2 is a member of the chemokine family also known as monocyte chemoattractant protein-1. The concentration of CCL2 return to baseline within 2 h of symptom onset [90].

Platelet activation factor (PAF) is a potent phospholipid-derived mediator implicated in platelet aggregation and secreted by mast cells, monocytes and tissue macrophages. Few reports assessed the concentrations of PAF or the enzyme responsible for its rapid degradation, the platelet-activating factor acetylhydrolase (PAF-AH), in human anaphylaxis, and found increased circulating PAF levels with PAF-AH activity inversely correlated with the reaction severity. PAF may be determined using the ^3^H-scintillation proximity assay system. However, these biomarkers are unattractive candidates for routine clinical use due to special sample requirements and very short half-life (ranging from 3 to 13 min for PAF) [54,91,92]. 

Cysteinyl leukotrienes are produced from arachidonic acid by a variety of cells, including mast cells, basophils, and macrophages, and represent another class of potential mediators in anaphylaxis. Some reports show that levels of some of their products, such as leukotriene E4 (LTE4) and 9*α*, 11*β*-prostaglandin F2 (9*α*, 11*β*-PGF2), are increased at the onset of anaphylaxis, but, similar to histamine, they are measured in 24-h urine collection. Therefore the sensitivity might be low [2,93]. Urinary LTE4 and 9α, 11β-PGF2 concentrations may be determined by enzyme immunoassays [94]. Like histamine, elevated serum levels of PGD2 are difficult to detect due to the rapid clearance from the systemic circulation. 2,3-Dinor-9*α*,11*β*-PGF2 is a more predominant PGD2 metabolite in urine than 9*α*,11*β*-PGF2 [95].

Inflammatory cytokine biomarkers, such as TNF-*α*, IL-6, and IL-1*β*, can be increased in serum of patients with anaphylaxis and cytokine storm-like reactions, but their sensitivity or specificity is still not demonstrated [2]. A recent study comparing baseline and reaction samples in perioperative anaphylaxis revealed IL-6 and CCL2 as potential biomarkers [96].

Another study performed to characterize serum biomarkers during anaphylaxis in the emergency department patients identified elevated IL-6 and IL-10 levels, which could be a unique signature of anaphylaxis. It is necessary to further compare the utility of such biomarkers to tryptase, the common biomarker for acute anaphylaxis. No other significant differences were detected for other cytokines, such as IL-1B, IL-2, IL-4, IL-8, IL-12, IL-13, IL-18, TNFα, and for complement C5a or adhesion molecule ICAM-1 [97]. Activation of the coagulation/fibrinolytic system usually appears together with the activation of the complement system, which is considered a triggering mechanism in severe anaphylaxis. C3a, C4a and C5a (referred classically as anaphylatoxins) are considered biomarkers of complement activation [98,99,100].

Despite mast cell heparin may activate the plasma contact system in anaphylaxis, there are no available assays to quantify it directly. Anti-Xa may be quantified using a chromogenic assay and represents an indirect measure of plasma heparin, but the method does not have high sensitivity [101].

Regarding the quest for future endothelial biomarkers in anaphylaxis, a recent proteomic and biological analysis of an in vitro human endothelial system was performed [100].

Two different molecules (Regulator of calcineurin 1 and Fibroblast growth factor-inducible molecule 14) may be involved in the modulation of histamine-induced endothelial barrier disfunction in anaphylaxis, as revealed in cellular and murine experiments, but their role as biomarkers is not established [102,103]. Because endothelial cells increase the miR-21-3p intracellularly and release it in response to anaphylaxis, this researched microRNA may be a future candidate biomarker [104].

To sum up, in the overview Table 3 are presented selected candidate biomarkers for anaphylaxis in obstetrics with potential usefulness in acute care settings. 

### 4.3. Biomarkers for IgE Sensitization Assessment 

IgE antibodies play the most important role in triggering an anaphylactic reaction. Physiologically, they are the weakest isotype represented in circulation (50–200 ng/mL of blood), and their concentration is significantly increased in people with atopy [28,29]. IgE binds to the high-affinity Fc*ε*RI receptor mainly on the surface of basophils and mast cells [28]. During the allergen exposure, the link between specific IgE and Fc*ε*RI activates these cells, and causes the immediate release of preformed mediators such as histamine, various proteases, and later on the release of *de novo* mediators such as leukotrienes, prostaglandins, cytokines [28,29].

The most frequent triggers of IgE-mediated anaphylaxis in pregnancy are drugs, foods and insect venoms, but other elicitors are mentioned in the scientific literature. As in nonpregnant patients, venom from stinging Hymenoptera insects (honey bees, wasps, fire ants) and ingestion of foods, such as shellfish, peanuts, or tree nuts, can cause anaphylaxis during pregnancy.

Antibiotics commonly reported as triggers of anaphylaxis in pregnant women are administered either before cesarean delivery for prophylaxis against surgical site infection or during labor for prophylaxis against early-onset group B streptococcal infection. These medications include penicillins and cefalosporins. Medications used for neuraxial and general anesthesia may also cause anaphylaxis. Neuromuscular blocking agents, such as suxamethonium, are a common cause. Oxytocin, intravenous iron, Laminaria osmotic dilators, misoprostol and natural rubber latex were also reported as triggers of anaphylaxis in preganancy [6,11].

Drug specific IgE determination in serum may be performed using singleplex fluorescence enzyme immunoassay with capsulated cellulose polymer solid-phase for beta-lactam antibiotics, neuromuscular-blocking agents, chlorhexidine, opioid or enzyme-enhanced chemiluminescence immunoassay with liquid-phase allergens for beta-lactam antibiotics. In pregnant patients with beta-lactam induced anaphylaxis, clearly positive serum specific IgE can be useful for avoiding both allergy skin tests and drug provocation tests. Serum specific IgE quantification can be used for a limited number of drugs in the perioperative setting, reported sensitivity and specificity are very good for specific IgE for latex and chlorhexidine, but show great variation for neuromuscular-blocking agents and opioids [2,56,105]. 

Hypersensitivity adverse reactions, including features of anaphylaxis, have occasionally been reported after administration of anti-D immunoglobulin in pregnant women. In an experimental assay, specific IgE to an antigenic contaminant, but not anti-D itself, was detected [106,107].

In progestogen hypersensitivity, typically occurring in women in childbearing years with a spectrum of symptoms ranging from dermatitis, urticaria to anaphylaxis, associated with exogenous progestin exposure (contraceptive medicines, in vitro fertilization therapy) or endogenous progesterone from progesterone surges during the luteal phase of the menstrual cycle and pregnancy, the most commonly accepted mechanism is IgE-mediated sensitization. Progesterone specific IgE biomarker may be determined by an enzyme-linked immunosorbent assay (ELISA) [108]. Changing levels of progesterone and/or prolactin were also suggested as possible predisposing factors in breastfeeding induced anaphylaxis, sometimes in association with the administration of nonsteroidal anti-inflammatory drugs, which are potential non-IgE-mediated anaphylaxis triggers [109,110,111,112,113]. 

Specific IgE against natural food extracts, Hymenoptera (bees, wasps) venom extracts and latex determined in the serum using singleplex or multiplex immunoassays, preferable with cross-reactive carbohydrate determinant (CCD) inhibition, reduce the need for high-risk tests in pregnancy. Moreover, molecular biomarkers, native or recombinant, are more useful in this purpose when used in the precision approach of component-resolved diagnostics [2,13,56,114].

Food anaphylaxis is associated with IgE sensitization to certain molecules from foods of animal and vegetal origin, such as seed storage proteins (2S albumins, 7S vicilins, 11S legumines), nonspecific lipid transfer proteins (nsLTPs), fish parvalbumins, shellfish tropomyosins, and wheat *ω*-5-gliadin in cofactor-enhanced food allergy. PR-10- and profilin-induced systemic reactions are facilitated by proton-pump inhibitors, ingestion of large amounts of unprocessed foods, and fasting. Severity risk attributed to specific molecules may vary according to other factors such as geographic variations, degree of allergen exposure, cross-reactivity, co-sensitizations, and cofactors, such as exercise, alcohol, nonsteroidal anti-inflammatory drugs [2,115,116,117].

Serum specific IgE antibodies for non-primate mammalian carbohydrate galactose-*α*-1,3-galactose (*α*-Gal) are associated to *α*-Gal syndrome with delayed allergy to red meat manifested usually as anaphylaxis after ingestion of beef, pig or lamb meat. IgE-mediated drug allergy in this syndrome was reported for some therapeutic monoclonal antibodies, such as cetuximab, snake antivenom, and varicella-zoster vaccine, but there are also risks for colloid plasma volume substitutes [118]. 

Some experts consider that, compared with non-pregnant patients in whom neuromuscular blocking drugs are common drug triggers, allergic reactions in parturients mostly occur to antibiotics, uterotonics and infusion of colloids [119]. 

Vaginal capsules may also represent a hidden source of exposure to the *α*-Gal epitope, anaphylaxis being reported in a female patient with recurrent episodes of anaphylaxis after eating red meat and after application of an intravaginal capsule with gelatin cover with collagen of porcine origin [120].

The bovine thyroglobulin (Bos d) *α*-Gal carrying molecule is the molecular biomarker used in the singleplex fluorescence enzyme immunoassay with capsulated cellulose polymer as solid-phase for assessing IgE sensitization to *α*-Gal [118].

Some case reports revealed that bovine serum albumin (BSA) may be a causative agent in anaphylaxis, even severe, after standard intrauterine insemination or in vitro fertilization, when added to the culture medium of spermatozoids [121]. BSA contained in culture media used in artificial insemination is a significant anaphylaxis risk factor in patients allergic to cats. As cat allergen biomarker Fel d 2 may predict cross-reactivity to nonhuman mammalian serum albumins, such as domestic cattle Bos d 6, assessment of IgE sensitization to these molecular allergens in cat-allergic patients, by singleplex or multiplex IgE immunoassays, could be meaningful in this context [122]. Several case reports of human seminal plasma allergy in women sensitized to dog prostatic kallikrein biomarker Can f 5, an arginine esterase allergen from dog urine and dander, were reported [57,123,124,125]. 

Anaphylaxis following laminaria insertion occurs rarely, but may be life-threatening. Laminaria tents, prepared from natural sea kelp, are used prior to elective termination of pregnancy to achieve cervical dilatation. Laminaria consists principally of a glucan called laminarin (active ingredient in dilatation by modifying uterine prostaglandin metabolism or osmotic pressure) and trace amounts of protein. Specific IgE to laminaria extract may be measured using ELISA, but the allergenic component of laminaria leaf has not been identified [126].

Few case reports of Hymenoptera sting-induced hypersensitivity reactions are reported in the obstetric literature, focused especially on anaphylaxis [127,128]. Recommended biomarker molecular allergens of genuine (species-specific) IgE sensitization to hymenoptera venoms include Api m 1 and Api m 10 for honeybee (*Apis mellifera*) venom, Ves v 1 and Ves v 5 for common wasp (*Vespula vulgaris*) venom, and Pol d 5 for paper wasp (*Polistes dominula*) venom, used in singleplex and multiplex immunoassays [129].

Because latex allergy may be a cause of anaphylaxis in pregnancy, even though its incidence has significantly decreased in the last decade, molecular diagnosis by singleplex or multiplex immunoassays uses biomarkers of genuine latex allergy, such as rubber elongation factor Hev b 1, small rubber particle protein Hev b 3, acidic protein Hev b 5 and hevein precursor Hev b 6, while profilin Hev b 8 and CCD biomarkers reveal cross-reactivity and asymptomatic sensitization [2,130,131,132]. In a case of intraoperative IgE-mediated anaphylactic shock due to latex during elective cesarean section in a 38th week of pregnancy woman, plasma tryptase levels at 1 and 6 h after the episode of hypotension were within normal range, but with high levels of specific IgE to latex [133].

In clinical practice, oxytocin may constitute a risk factor for anaphylaxis, asthma and cardiologic side effects in delivering women [134,135,136].

There have been a number of reports of anaphylactic reaction related to synthetic oxytocin administration, some authors advocating the possibility of cross-reactivity with latex. There is homology in the protein sequence of oxytocin and latex patatin-like allergens Hev b 7.01 and Hev b 7.02 [136] but these molecular components are not available in current commercial IgE immunoassays.

A couple of life-threatening anaphylactic reactions with onset a few minutes after the infusion of oxytocin in women sensitized to latex allergens were reported during caesarian section under spinal anaesthesia in the delivery room [134]. Moreover, it has been also demonstrated that oxytocin, the hypothalamic neuropeptide which regulates labor-associated uterine contractions and milk discharge during lactation, has functional receptors on human airway smooth muscles, and pro-inflammatory cytokines, such as TNFα and IL-13 are known to be involved in asthma pathogenesis [136]. The airway inflammation in asthmatic women may be an independent risk factor for bronchoconstriction after infusion of oxytocin during delivery, apart from anaphylaxis.

Therefore, special care is needed in delivering women with asthma and latex allergy regarding a latex-free setting, appropriate pharmacotherapy and the use of oxytocin-alternative agents in order to reduce the risk of bronchoconstriction or anaphylaxis [137,138].

Moreover, in obstetric surgery, oxytocin and vasopressin can induce side effects such as negative inotropic and chronotropic effects, vasodilatation and low blood pressure, which may simulate anaphylaxis with consequent intraoperative diagnosis problems, probably underestimated in real life. Significant cardiorespiratory side effects of oxytocin are probably related to dosage and/or modality of infusion [138,139].

There is a need to differentiate side effects of oxytocin or vasopressin from allergic adverse reactions, but this is not always easy, having also in mind that biomarkers of latex IgE sensitization to patatin-like protein allergens are not available commercially. In some patients, cardiopulmonary symptoms may be due to side effects of oxytocin, but circumstances with urticaria and angioedema are more consistent with an IgE-mediated hypersensitivity reaction. In addition, the cardiopulmonary effects of oxytocin may amplify the signs and symptoms of anaphylaxis [140,141].

Basophil activation test (BAT) using flow cytometry has emerged as a sensitive marker used to detect clinically relevant triggers in anaphylaxis, being useful in drug allergy, food allergy and Hymeoptera venom allergy. Several studies have reported the diagnostic accuracy of BAT for allergy to a range of drugs, including beta-lactams, fluoroquinolones and neuromuscular blocking agents [2].

Moreover, positive BAT results to galacto-oligosaccharides (GOS) in milk formulae and food products were reported in rare cases of anaphylaxis in Asian pregnant and lactating mothers [142].

## 5. Management of Anaphylaxis

**Figure 2:**
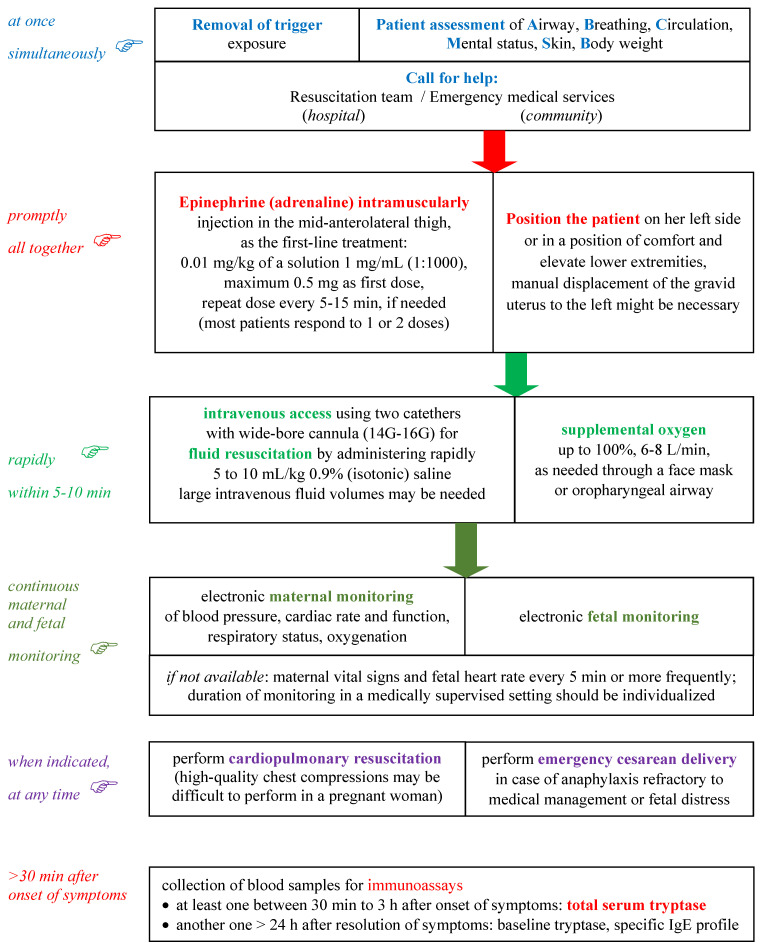
Management of anaphylaxis in pregnancy, according to World Allergy Organization guidelines [8,10].

The treatment of anaphylaxis of pregnant patients in acute care settings is basically the same as that in non-pregnant ones and it is not currently guided by biomarkers. Moreover, there are no anaphylaxis biomarkers to help identify the onset of biphasic anaphylaxis in clinical settings [143]. Pregnant patients with anaphylaxis may require emergency cesarean delivery to avoid fetal hypoxemia and prevent severe fetus damage, but currently, there are no validated molecular biomarkers for this indication [13]. The negative neurologic outcomes of the fetus are mostly due to delayed caesarean delivery or inadequate doses of adrenaline during anaphylaxis [12,16]. Figure 2 presents the management of anaphylaxis in pregnancy, including the optimal moment for blood specimens collection for biomarkers in the timeline of the emergency procedure.

In a parturient patient with anaphylaxis, it is important to follow the critical steps presented in Figure 2. Removing exposure to the trigger is very important if possible e.g., interrupt the administration of parenteral diagnostic/therapeutic agent likely involved, with a rapid assessment of the patient (airways, breathing, circulation, mental status, skin, bodyweight) as soon as anaphylaxis is clinically diagnosed or strongly suspected. Simultaneously calling for help from the emergency medical services in the community or the resuscitation team in a hospital setting is a must. The involvement of a multispecialty resuscitation team: anesthesiologist, obstetrician and neonatologist, is needed.

Epinephrine (adrenaline) intramuscularly injected into the vastus lateralis of the quadriceps (antero-lateral thigh) is the first-line treatment of anaphylaxis. The recording of epinephrine posology (dosing and time of administration) is required. There are no absolute contraindications to epinephrine in the setting of anaphylaxis. No laboratory biomarkers are needed for the decision of epinephrine usage. Ephedrine is a less potent vasoconstrictor and bronchodilator than epinephrine and its administration has been associated with increased fetal distress and acidosis [8,10,19,144]. Epinephrine for parenteral use should be rapidly available for pregnant women with potential risk of anaphylaxis [13]. Intramuscular epinephrine is generally well tolerated, but administered by intravenous route as initial treatment may potentially induce severe arrhythmias, even fatal as a result of bolus administration [8]. Repeated doses of epinephrine may be needed if symptoms are refractory to the first one.

Putting the patient safely in a semi-recumbent position on her left side or in a position of comfort and elevate lower extremities is required. Positioning on the back of the pregnant patient may lead to compression of inferior vena cava by the gravid uterus. A comfort position is needed if there is respiratory distress and/or vomiting. Suddenly sitting upright or standing may be associated with cardiac arrest, due to the empty inferior vena cava/empty ventricle syndrome, with fatality within seconds. The manual displacement of the gravid uterus to the left might be necessary to prevent added positional hypotension [8,50].

Intravenous access using two catheters with a wide-bore cannula and fluid resuscitation with intravenous fluids are needed. Oxygen therapy should also be considered in any patient with symptoms of anaphylaxis. Vasopressors, such as dopamine, can also be considered if epinephrine injections and volume expansion with intravenous fluids fail to alleviate hypotension. An adequate intravascular volume repletion is important to treat hypotension and maintain uteroplacental perfusion. The rate of infusion should be titrated against blood pressure, cardiac rate and function ascertained by continuous noninvasive monitoring, and urine output. 0.9% (isotonic) saline is a favored crystalloid. Glucose-containing solutions should be avoided, because a large glucose bolus can reduce umbilical cord pH and induce neonatal hypoglycemia. Significant volumes of intravenous fluids may be needed to promptly reverse hypotension, maintaining a minimum maternal systolic blood pressure of 90 mmHg for an adequate uteroplacental perfusion and fetal oxygenation since uterine blood flow is not autoregulated. Supplemental oxygen is also important because epinephrine can potentially worsen the ventilation/perfusion ratio due to its alpha_1_-adrenergic vasoconstrictor effect [8,144].

Second-line medications in anaphylaxis may be administered only after the intramuscular injection of epinephrine. There is frequently a concern that their administration can delay important more urgent measures such as repeated administration of intramuscular epinephrine. An inhaled selective short-acting beta_2_-agonist, such as salbutamol, may be administered with a jet nebulizer for additional relief of bronchospasm. A systemic second-generation H_1_ antihistamine with relatively rapid onset of action, such as cetirizine, although it is not life-saving, may be used for additional relief of pruritus and urticaria. The first-generation H_1_ antihistamines can cause hypotension at rapid intravenous administration and sedation which are counterproductive in anaphylaxis. Intravenous cetirizine, the only second-generation parenteral H_1_ antihistamine, available in some countries, may be recommended in the treatment of acute urticaria [145], but as any other H_1_ antihistamine should never replace intramuscular epinephrine as first-line therapy in anaphylaxis. An injectable H_2_ antihistamine, also not life-saving in anaphylaxis, is sometimes used. But it should be kept in mind that there are case reports of anaphylaxis to ranitidine in the parturient. A parenteral glucocorticoid, such as hydrocortisone or methylprednisolone succinate, has no immediate life-saving benefits, it is administered with the intention to prevent biphasic or protracted anaphylaxis. But there is increasing evidence that systemic corticosteroids may be of no benefit in the acute management of anaphylaxis due to their slow onset of action and potential detrimental adverse effects [146,147]. Therefore their routine use is becoming controversial [148,149]. A protracted anaphylactic reaction persists for at least four hours without clearly resolving completely, while biphasic anaphylaxis is characterized by an initial anaphylactic reaction followed by an asymptomatic period of one hour or more, and then a subsequent return of symptoms meeting the criteria for anaphylaxis without further exposure to the offending trigger [150,151].

## 6. Conclusions

Currently, mast cell tryptase remains the only standard laboratory test for anaphylaxis in acute care settings, while specific IgE molecules against many allergen triggers and their molecular components are also currently available in clinical practice to assess IgE sensitization. These biomarkers are useful because in vivo allergy tests cannot be performed on pregnant women in such a major medical emergency due to their additional potential risk of anaphylaxis.

Despite extensive research, other biomarkers for detecting anaphylaxis are still elusive. Candidate biomarkers will possibly expand the possibilities for a more accurate diagnosis and phenotype and endotype classification of anaphylaxis, due to their potential in improving diagnosis and management, with stratification of severity and risk prediction.

Identification and validation of novel valuable biomarkers along with the wide clinical availability of new generation immunoassays and cell-based assays such as BAT still represent unmet needs in the management of anaphylaxis in general and in obstetrics in particular. For the future outlooks concerning upcoming biomarkers and the acute management of anaphylaxis in pregnant women it is worth mentioning CCL2, a promising potential biomarker that seems less affected by the surgical setting, along with chymase and carboxypeptidase A3, and the possible role for combinations of mast cell tryptase and such novel biomarkers to augment sensitivity and specificity. In our opinion, the most promising molecular biomarkers of IgE sensitization are those related to immunoassays for component resolved diagnosis, due to their commercial availability and the range of microarray/macroarray formats.

A deeper understanding of the pathogenesis mechanisms of anaphylaxis, together with systems biology, proteomics and multi-omics approaches, may provide forthcoming clinically useful biomarkers or new biomarker signatures in anaphylaxis.

## Figures and Tables

**Table 1:** Criteria for the diagnosis of anaphylaxis from World Allergy Organization [8,9].

Anaphylaxis Is Highly Likely When Any One of the Following 2 Criteria Are Fulfilled:
1. Acute onset of an illness (minutes to several hours) with simultaneous involvement of the skin, mucosal tissue, or both (e.g., generalized hives, pruritus or flushing, swollen lips-tongue-uvula)
AND AT LEAST ONE OF THE FOLLOWING:
a. Respiratory compromise (e.g., dyspnea, wheeze-bronchospasm, stridor, reduced PEF, hypoxemia)
b. Reduced BP or associated symptoms of end-organ dysfunction (e.g., hypotonia [collapse], syncope, incontinence)
c. Severe gastrointestinal symptoms (e.g., severe crampy abdominal pain, repetitive vomiting), especially after exposure to non-food allergens
2. Acute onset of hypotension ^a^ or bronchospasm ^b^ or laryngeal involvement ^c^ after exposure to a known or highly probable allergen ^d^ for that patient (minutes to several hours), even in the absence of typical skin involvement.

Legend: PEF, Peak expiratory flow, BP, blood pressure. ^a^ Hypotension defined as a decrease in systolic BP greater than 30% from that person’s baseline, OR. i. Infants and children under 10 years: systolic BP less than (70 mmHg + [2 × age in years]). ii. Adults and children over 10 years: systolic BP less than <90 mmHg. ^b^ Excluding lower respiratory symptoms triggered by common inhalant allergens or food allergens perceived to cause “inhalational” reactions in the absence of ingestion. ^c^ Laryngeal symptoms include stridor, vocal changes, odynophagia. ^d^ An allergen is a substance (usually protein) capable of triggering an immune response that can result in an allergic reaction. Most allergens act through an IgE-mediated pathway, but some non-allergen triggers can act independently of IgE (for example, via direct activation of mast cells).

**Table 2:** Anaphylaxis triggers reported in pregnancy and involved general immunologic/nonimmunologic mechanisms.

Triggers	References	Mechanism Involved	Effector Cells	Important Mediators
penicillins, foods, venom, latex	[2,6,8,11]	IgE-dependent	mast cell/basophil	histamine, tryptase, chymase, carboxypeptidase
iron (intravenous)	[2,41,42]	Complement system	mast cells	histamine, PAF, C3a, C5a
neuromuscular blockers	[2,6,8,11]	Mast cell/basophil activation MRGPRX2; IgE-dependent	mast cells	histamine, tryptase, chymase, heparin, PAF

Legend: IgE = Immunoglobulin E; Complement components C3a, C5a = anaphylatoxins; PAF = Platelet-activating factor; MRGPRX2 = Mas-Related G-Protein Coupled Receptor Member X2.

**Table 3:** Selected candidate biomarkers with potential applicability in the diagnosis of anaphylaxis in acute settings.

Candidate Biomarker	Method of Detection	Comments	References
serum/plasma histamine	enzyme immunoassay	short half-life	[53,55]
serum/saliva carboxypeptidase A3	enzyme-linked immunoassay	half-life longer than tryptase	[55,90]
serum chemokine ligand CCL2	sandwich enzyme immunoassay	glycosylation influences half-life	[55,91]
serum interleukin IL-6	sandwich enzyme immunoassay	inflammatory cytokine	[97,98]

## Data Availability

Not applicable.
